# A Rational Approach to Pharmacotherapy in Pregnancy

**DOI:** 10.1002/jcph.70145

**Published:** 2025-12-17

**Authors:** Gregory W. Kirschen, Kevin Watt, Ahizechukwu C. Eke

**Affiliations:** ^1^ Department of Obstetrics and Gynecology Division of Maternal Fetal Medicine Hospital of the University of Pennsylvania Philadelphia PA USA; ^2^ Pediatrics Department University of Utah Health Salt Lake City UT USA; ^3^ Department of Gynecology and Obstetrics Division of Maternal Fetal Medicine The Johns Hopkins Hospital Baltimore MD USA

**Keywords:** pharmacokinetics, pharmacodynamics, placental transfer, physiological changes of pregnancy

## Abstract

Most pregnant individuals are exposed to at least one medication, whether prescription or over the counter, during pregnancy. Despite the ubiquity of medication use in pregnancy, there remains no standardized framework to guide clinicians in selecting the most appropriate pharmacotherapy that balances maternal needs with fetal safety. This gap contributes to variability in prescribing practices and uncertainty in clinical decision making. In this article, we propose a structured schema for evaluating and selecting drug therapy during pregnancy. Our approach emphasizes careful consideration of maternal and fetal factors, integration of the unique physiologic changes of pregnancy, and systematic appraisal of the best available evidence. Recognizing the frequent absence of robust pharmacokinetic and safety data, we also provide pragmatic principles and rules of thumb to guide clinicians in estimating the likelihood of placental transfer and potential fetal exposure. This framework is designed to support clinicians in making more informed, transparent, and evidence‐based decisions while also identifying areas for future research.

## Introduction

Medication use in pregnancy is ubiquitous, with the vast majority (up to 97%) of pregnant women taking at least one medication during pregnancy, and a similar number reporting at least one medication used in the first trimester.^1^, ^2^ Off‐label prescribing in pregnancy is also common, given financial constraints, practical considerations, and lack of incentives for pharmaceutical companies to seek Food and Drug Administration (FDA) labeling in pregnancy of drugs already on the market.^3^ Moreover, certain over the counter (OTC) medications that have been linked to teratogenic effects, such as ibuprofen, are used by 15%–18% of pregnant women.^4^, ^5^

Limitations exist in our body of evidence regarding the safety and efficacy of both OTC and prescription medications in pregnancy. This poses challenges when attempting to weigh risks and benefits of a particular medication that may, for instance, have known maternal benefit but unknown fetal risk. Additionally, absorption, distribution, metabolism, and excretion of drugs may be altered given the physiological changes of pregnancy, which we detail below.^6^

Given the complexities of decision making surrounding appropriate selection of medications in pregnancy, it is thus critical that obstetricians and other prenatal care providers understand the basic principles of pharmacology and use this in conjunction with the best available evidence to make rational drug decisions to safeguard both mother and fetus. This article thus serves to provide a basic framework for clinicians and researchers in conceptualizing an approach to drug therapy in pregnancy. Of note, lactational pharmacology is beyond the scope of the present work.

## General Considerations on Pharmacotherapy

When deciding to initiate pharmacotherapy in pregnancy, we advise considering the indication for treatment, and then weighing the maternal benefits and risks, and fetal benefits and risks. Once this balanced deliberation has taken place, a shared decision can be made between the clinician and the patient regarding the safest and the most efficacious drug to be employed for a given condition.

### Choosing Treatment When Maternal/Fetal Risks Are Known

Most prescriptions in pregnancy are intended for maternal benefit. The first step in deciding on appropriate therapy in this situation is to arrive at the optimal treatment for the pregnant person and then balance maternal benefits against fetal risks. For instance, take the case of a pregnant woman coming into the office with a suspected uncomplicated acute bacterial sinusitis. One would consider prescribing two categories of medications: (1) those for symptomatic relief, and (2) those for definitive treatment. Medications falling into the first category would include intranasal saline, intranasal ipratropium bromide, oral decongestants, intranasal decongestants, antihistamines, and mucolytics. For definitive treatment, one would consider a course of antibiotics. The decision regarding which antibiotic to select would be guided by predicted efficacy, duration of therapy, local resistance patterns, as well as any contraindications such as penicillin allergy. In the case of uncomplicated acute bacterial sinusitis, a reasonable first‐line agent would be amoxicillin or amoxicillin clavulanate. If the patient does not improve after an appropriate duration of first‐line antibiotic therapy, then second‐line agents would include higher dose amoxicillin clavulanate, a fluoroquinolone, a third‐generation cephalosporin plus clindamycin, or doxycycline.^7^ For each of these options, one would also consider potential maternal risks or serious adverse effects of treatment, including allergic reactions, tendinopathy/tendon rupture in the case of fluoroquinolones, and hepatotoxicity and photosensitivity in the case of doxycycline.^8^, ^9^, ^10^ One would next rule out certain classes of medications based on unacceptably high fetal risk.

### Approach to Treatment When Drugs Are Known to be Teratogenic and Contraindicated

When considering drugs for maternal benefit that may have off‐target fetal effects, important factors that will weigh into decision making include trimester of pregnancy, anticipated dosage and duration of drug use, and occasionally the sex of the fetus for drugs that may interfere with sexual differentiation (e.g., spironolactone). In the case of the woman with uncomplicated acute bacterial sinusitis, medications are sorted into three broad categories: known to be safe in pregnancy, known to be unsafe in pregnancy, or insufficient evidence of safety in human pregnancies. Based on these considerations, one determines that all symptomatic medications listed above are safe from a fetal perspective, with the possible exception of pseudoephedrine, which through its predominantly α‐adrenergic stimulant effect, has been theorized to cause uterine artery constriction and fetal hypoxia with resultant modest increases in risk of vascular‐disruption gastroschisis, intestinal atresia, and hemifacial microsomia.^11^ On the other hand, a follow‐up large‐scale human study failed to convincingly demonstrate signal for fetal harm.^12^ For this reason, local delivery of decongestants is preferred over oral formulations to minimize risk of systemic absorption, a general principle of pharmacology in pregnancy for agents with potential fetal risk.

One then considers antibiotic choice, noting that penicillin derivatives, cephalosporins, and clindamycin have all been successfully used in pregnancy and are deemed safe from a fetal perspective. One finally contemplates fluoroquinolones and doxycycline, and notes that these medications have known teratogenic effects including permanent dental discoloration, and thus would be considered relatively contraindicated in pregnancy.^13^, ^14^ For a comprehensive and easily accessible resource of other commonly used drugs and fetal risk, the reader is directed to Briggs Drugs in Pregnancy and Lactation.^15^

Note that few medications are absolutely contraindicated in pregnancy, and occasionally scenarios will arise in which a medication critical for maternal health is one that confers known fetal harm. Examples include warfarin for patients with a mechanical heart valve, hydroxyurea for sickle cell disease (SCD), and chemotherapy for malignancy.

Warfarin, a vitamin K antagonist, is the only anticoagulant approved and guideline recommended for the prevention of thromboembolism in patients with mechanical heart valves, as it performs superiorly to heparin derivatives and direct oral anticoagulants, DOACs (the latter of which are contraindicated in pregnancy, regardless).^16^ Warfarin is known to cross the placenta and is associated with fetal skeletal abnormalities including nasal hypoplasia and facial and long bone anomalies, as well as stillbirth.^17^, ^18^ This is balanced against the higher risk of composite adverse maternal outcomes, including death, thromboembolism, and valve failure with the use of low molecular weight heparin (LMWH, 16%), compared to that of warfarin (5%) in the pregnant population.^19^

SCD is a hemoglobinopathy characterized by a variant in the structure of the β‐globin chain of hemoglobin (resulting in production of HbSS instead of HbA) leading to sickling of red blood cells in the microvasculature under hypoxic conditions in patients harboring two variant copies of the abnormal beta globin gene.^20^ Hydroxyurea increases production of fetal hemoglobin (HbF) and limits the production and polymerization of abnormal HbSS hemoglobin.^21^ This drug has been shown to decrease hemolysis and need for transfusion and to decrease vaso‐occlusive crises in patients with SCD. In 2007, the National Toxicology Program and the National Institute of Environmental Health Science reviewed animal and human data regarding the reproductive safety of hydroxyurea and concluded that there were insufficient human data to support safety, and that animal data raised concerns for congenital anomalies and fetal growth restriction.^22^, ^23^ Subsequent work examining human pregnancy outcomes among recipients of hydroxyurea demonstrated in multivariate analysis that there was no association between hydroxyurea use and miscarriage or stillbirth with use limited to time of conception compared with no use, however there was higher risk of these outcomes with hydroxyurea use at conception and during pregnancy compared to no use, after adjusting for covariates.^23^ Despite these reservations, scenarios arise in which the benefits of SCD treatment with hydroxyurea to prevent severe maternal morbidity may outweigh known fetal risks. Another general principle in pharmacotherapy in pregnancy that this scenario highlights is that untreated severe maternal medical disease in pregnancy in and of itself may lead to fetal harm, thus risks of fetal harm with treatment must also be weighed against risks of fetal (in addition to maternal) harm with no treatment.

### Approach to Maternal Malignancy

Another situation that complicates approximately 0.1% of pregnancies and poses a treatment dilemma is maternal malignancy.^24^ The most common malignancies encountered during pregnancy include breast cancer, cervical cancer, melanoma, lymphomas, and leukemias.^24^ Specific treatment regimens vary widely across these different cancer types and even within organ‐specific cancers, depending on histology, stage, and subtype, and an excellent comprehensive review of chemotherapeutic class and teratogenic effects has been previously performed.^25^ Still, several general principles in the approach to cancer pharmacotherapy in pregnancy can be derived. Many cancer treatments are compatible with pregnancy and in most cases benefits of treatment outweigh risks. In the first 12 weeks of gestation during organogenesis, the overall risk of chemotherapy leading to congenital malformations is between 10 and 20%, versus 3% after 12 weeks’ gestation.^26^ In the second and third trimesters, the most common adverse fetal effects of chemotherapy encountered include fetal growth restriction and prematurity.^27^ Therefore, monitoring of fetal growth with serial ultrasounds is prudent. Although maternal malignancy and chemotherapy exposure in pregnancy have been linked to alterations in neonatal functional brain organization, clinically significant differences in neurocognitive outcomes have not been conclusively demonstrated, especially when accounting for prematurity.^28^, ^29^

Certain chemotherapeutic agents, such as methotrexate (a folic acid antagonist) and cyclophosphamide (a DNA alkylating agent and cross‐linker), are known to cause miscarriage or stillbirth as well as fetal malformations through cytotoxic effects on rapidly dividing cells. In surviving neonates, methotrexate and cyclophosphamide exposure have been associated with anomalies of the central nervous, craniofacial, and skeletal systems.^30^, ^31^ Therefore, these agents are considered absolutely contraindicated in ongoing intrauterine pregnancies, and patients should be counseled on the option for termination of pregnancy or iatrogenic preterm delivery, depending on the gestational age and patient's goals of care, if suitable alternative chemotherapeutic agents are unavailable or inferior, and if treatment cannot be safely deferred until after delivery. Finally, delivery considerations include timing around chemotherapy administration, as delivery in close proximity to chemotherapy administration (generally within 2–3 weeks) can be associated with complications such as maternal and fetal immunosuppression and bone marrow suppression (predisposing to infection in mother and fetus, and postpartum hemorrhage in mother).^24^, ^25^ Multidisciplinary care for the patient involving the primary oncology team, anesthesiology, neonatology, and maternal fetal medicine is essential.

Many other examples abound of maternal medical diseases for which treatment of the condition in pregnancy is often outweighed by known risks to fetus, such as psychiatric medications used for maternal mental illness (e.g., lithium for bipolar disorder) and certain biologic agents for autoimmune diseases. In cases in which a known teratogen is under consideration for pharmacotherapy, it is important not only to weigh benefits to maternal health against risks to fetal health, but also to formulate a plan for tracking potential adverse fetal effects, which may include an early anatomical survey, serial growth ultrasounds, and/or antenatal fetal surveillance. Enrollment of the patient into a drug safety monitoring registry can also be prudent, as this can provide longitudinal neonatal and childhood outcome data for pregnancies with known teratogen exposures. On the other hand, in cases in which standard of care treatments for maternal benefit are deferred by the patient in the interest of fetal health, it is important to formulate a second‐ and third‐line therapy plan, and to consider whether a threshold exists for reconsidering first‐line therapy (e.g., maternal decompensation) especially in cases in which a poor maternal health outcome would likely also result in a poor fetal outcome. In each of these scenarios, it is imperative to engage in shared decision making with the patient and her family.

### Medications Intended for Fetal Benefit

Finally, situations may arise in which drugs that may be associated with risk to the mother are prescribed in pregnancy for fetal benefit alone. One example is antiarrhythmic medications including digoxin, sotalol, and flecainide for the treatment of fetal tachyarrhythmias.^32^ In such cases, the benefits to the fetus, such as controlling fetal arrhythmia and circumventing fetal heart failure and hydrops fetalis may outweigh the potential side effects to the mother, including dizziness, gastrointestinal upset, or blurred vision. Of note, serious adverse maternal effects such as iatrogenic maternal arrhythmia induced by these agents, must be considered and the patient must be appropriately counseled and consent to the use of such drugs which confer potential fetal benefit with only maternal risk. Another example is the use of sirolimus for treatment of fetal tumors including sacrococcygeal teratoma and cardiac rhabdomyoma as well as vascular malformations in utero.^33^, ^34^, ^35^ In these cases, the hypervascularity of the tumor or vascular malformation that the fetus is effectively transfusing can lead to high output cardiac failure, hydrops fetalis, and fetal demise. Treatment with sirolimus, an mTOR inhibitor which blocks cell growth and proliferation and crosses the placenta, has been shown to improve fetal outcomes in these rare conditions.^33^, ^34^, ^35^, ^36^ When considering use of this agent, the potential maternal risks (immunosuppression and secondary malignancy) must be carefully weighed against the anticipated benefits to fetal well‐being, in accordance with maternal wishes, values, and risk tolerance.

**Figure 1:**
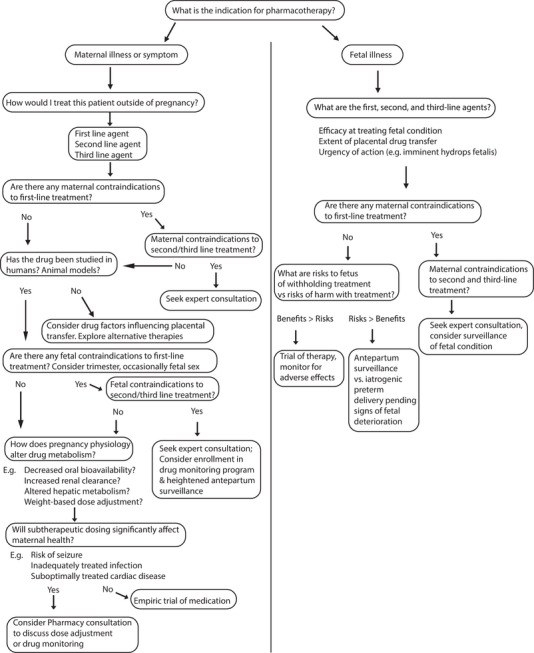
Decision tree for considering pharmacotherapy in pregnancy.

Figure 1 provides a decision tree that can be used when contemplating pharmacotherapy in pregnancy, whether for maternal or fetal benefit.

## Principles of Pharmacology in Pregnancy

To understand how pregnancy affects drug absorption, distribution, metabolism, and excretion (pharmacokinetics) and how drugs impact pregnancy and the body more generally including on target and off target effects (pharmacodynamics), it is worthwhile to consider physiological changes of pregnancy of different organ systems that affect these parameters. In this section, we provide a brief overview of how normal pregnancy physiology affects drug metabolism as well as principles of placental drug transfer to the fetus. Of note, while drug properties (e.g., molecular weight and polarity) can sometimes be used to heuristically gauge the extent to which a given drug may cross the placenta, we caution against attempting to predict placental transfer based solely on these properties. In many cases, drugs may be pumped into or out of the syncytiotrophoblastic and endothelial cell layers via active or facilitated transport channels, thus complicating any predictions of the extent to which a drug may cross the placental barrier.

### Effects of Changes in Maternal Body Composition on Pharmacokinetics

Several key adaptations occur in pregnancy that affect drug metabolism. Firstly, and most apparently, maternal weight increases in pregnancy. A normal weight individual is expected to gain 11–16 kg throughout pregnancy, with the average weight gain in the second and third trimesters increasing by roughly 0.3 to 0.7 kg per week.^37^ Therefore, drugs that require weight‐based dosing will typically need to be dose adjusted on a once per trimester basis, based on updated maternal weight. A common example of this is patients taking LMWH for prevention or treatment of venous thromboembolism. In patients for whom weight fluctuations deviate from the standard, or in those with renal impairment, or at those at especially high risk for thromboembolism, serial measurement of anti‐factor Xa levels may be considered.^38^ Regarding the composition of weight gain in pregnancy, this is important as it can affect factors such as drug volume of distribution and half‐life (both absorption and elimination half‐lives). In physiological pregnancy, weight gain is multifactorial, comprised of increased fluid volume (amniotic fluid and plasma), increased blood volume from erythrocyte production, and increased fat stores.^37^ Although amniotic fluid contributes to maternal weight gain, it does not always contribute to a drug's volume of distribution. For example, although the adjusted‐dose (therapeutic) regimen for LMWH is weight based, it distributes to total plasma volume, not total body water, and does not cross the placenta into amniotic fluid.^39^ Increased blood volume is comprised of expanded plasma volume and erythrocyte production; plasma volume increases (∼40%) disproportionately more than erythrocyte volume increases (∼18%–25%).^40^, ^41^ The increased body water in pregnancy affects changes in the volume of distribution only for hydrophilic drugs, compared to the relatively minor increase in body fat, which affects only lipophilic drugs. Thus, it is not easy to predict how pregnancy will affect drug half‐life or volume of distribution based solely on weight gain, as this will depend on drug factors including solubility, extent of placental transfer, as well as maternal factors such as degree of weight gain and volume status, in addition to a multitude of changes in other organ systems, described below.

### Gastrointestinal Adaptations that Affect Pharmacokinetics

From a gastrointestinal (GI) standpoint, gastric emptying is delayed in the first trimester due to the action of progesterone and relaxin, which act to impede smooth muscle cell contraction, however recent data demonstrate that gastric emptying time is equivalent to that of the non‐pregnant state in the second and third trimesters up until labor, when it is again slowed.^42^, ^43^, ^44^ Decreased esophageal smooth muscle tone also increases gastroesophageal reflux in pregnancy.^45^ These factors combined not only increase risk of nausea/vomiting in the first trimester, but also aspiration during cesarean delivery, and have implications for oral absorption of medications in the first trimester and at the end of pregnancy.

Factors that may limit oral absorption of medications in pregnancy include increased gastric pH (due to decreased gastric acid secretion), reflux esophagitis and nausea/vomiting, or hyperemesis gravidarum (HG) in severe cases. Recent studies have suggested a genetic component to HG including central sensitivity to the hormones GDF15 and IGFBP7 (previously attributed to beta hCG).^46^, ^47^ Orally administered drugs that are particularly pH sensitive include the antifungals ketoconazole and itraconazole, as well as the human immunodeficiency virus (HIV) drug atazanavir which requires a low stomach pH for optimal bioavailability.^48^, ^49^, ^50^ A special population to consider is those who have undergone weight loss surgeries such as roux‐en‐Y gastric bypass, sleeve gastrectomy, laparoscopic adjustable gastric banding, or biliopancreatic diversion with duodenal switch procedures, as well as patients entering pregnancy with pre‐existing gastroparesis such as that induced by long‐standing diabetes mellitus. Such patients are vulnerable to nutritional deficiencies and potentially variable absorption of drugs requiring gastric mixing. For instance, drugs that require gastric acidification for proper dissolution and absorption in the small intestine, such as levothyroxine, may be suboptimally absorbed in those with a history of gastric bypass surgery, compounded by the relative alkalinity of gastric pH during pregnancy.^51^, ^52^ Therefore, especially for drugs with narrow therapeutic windows or significant dose‐related toxicities, dose adjustments in consultation with a pharmacist and/or monitoring of serum drug levels may be warranted.

### Hepatic Metabolism and Elimination in Pregnancy

Another component of the GI system which changes in pregnancy is the hepatobiliary system. Due to estrogen‐induced delayed motility of the sphincter of Oddi, there may be delayed release of bile from the common bile duct into the duodenum upon stimulation by a lipid‐dense meal or orally ingested lipophilic drugs.^53^ As bile allows for solubilization and absorption of fats, fat‐soluble vitamins, and fat‐soluble drugs, delayed secretion of bile into the small bowel may limit the absorption of these compounds. Moving to the liver, this organ is responsible for producing many plasma proteins, the largest and most abundant of which is albumin. While most plasma proteins remain at stable concentrations in pregnancy, albumin concentrations drop due to hemodilution from the physiologically increased blood volume of pregnancy, and at least theoretically as a result of feedback inhibition from alpha fetoprotein (although this latter mechanism has not been conclusively demonstrated in humans), despite increased albumin synthesis in pregnancy.^54^, ^55^ While absolute serum drug levels remain relatively constant in pregnancy, the free fraction of certain highly albumin‐bound drugs (particularly neutral and acidic drugs) rises in pregnancy, especially in the third trimester.^56^, ^57^ Examples of such drugs include diazepam, phenytoin, phenobarbital, dexamethasone, propranolol, carbamazepine, tacrolimus, efavirenz, and clindamycin.^56^, ^57^ Likewise, the concentration of alpha‐1 acid glycoprotein, a serum protein which binds drugs with basic pH, decreases by ∼50% by the third trimester.^56^, ^58^ Thus, dose adjustments of highly protein‐bound medications may be required especially in later gestation.

Changes in hepatic metabolism represent a critical consideration for pharmacotherapy in pregnancy. As background, metabolism of drugs is divided into three phases, which generally result in the production of increasingly hydrophilic compounds that can more readily be excreted by the liver, gut, or kidney.^59^ Phase I metabolism refers to functionalization reactions primarily involving oxidation, reduction, and hydrolysis, that introduce or expose polar functional groups onto drugs, catalyzed by cytochrome P450 enzymes. The result is to increase the water solubility of drugs.^59^ Phase II refers to drug conjugation, whereby drugs are covalently linked to hydrophilic molecules such as glucuronic acid. Finally, in Phase III, drugs undergo transporter‐mediated elimination into bile, urine, or stool. In pregnancy, Phase I metabolism is characterized by an increase in CYP2C9 and a decrease in CYP2C19 activity.^60^ Thus, CYP2C9‐metabolized drugs (e.g., warfarin and phenytoin) may be eliminated more rapidly, while CYP2C19‐metabolized drugs (e.g., proton pump inhibitors and selective serotonin reuptake inhibitors [SSRIs]) may be eliminated more slowly.^60^ Regarding Phase II, the activity of UGT1A4, which metabolizes lamotrigine and labetalol, can increase ∼3–4‐fold in pregnancy.^61^, ^62^ For this reason, dosing frequency of labetalol must often be increased from twice daily to three times daily, and lamotrigine requirements often increase in pregnancy, to maintain therapeutic levels.

Other cytochrome enzymes whose activity is altered in pregnancy include CYP2D6 and CYP3A4 (increased), and CYP1A2 (decreased).^63^ Notable drugs metabolized by these hepatic enzymes include phenytoin, metoprolol, dextromethorphan, midazolam, and nifedipine (CYP2D6 and CYP3A4), and caffeine, theophylline, clozapine, tacrine, and propranolol (CYP1A2).^63^, ^64^, ^65^ Thus, patients taking these drugs in pregnancy, and especially those with compromised hepatic function at baseline, may require close therapeutic monitoring.

### Renal Metabolism and Elimination in Pregnancy

Apart from oral absorption and hepatobiliary metabolism, the renal system is a critical component of drug metabolism and elimination. As early as 5 weeks’ gestation, maternal systemic vasodilation occurs with subsequent decreased systemic vascular resistance, leading to increased renal blood flow and increased glomerular filtration rate (GFR).^66^ This is also in part mediated by relaxin‐induced increased nitric oxide synthesis, the effect of which is to increase renal vasodilation and plasma flow.^66^ In addition, changes in tubular function occur, with alterations in handling of glucose (leading to mild glucosuria in some women), increased excretion of amino acids, and a modest amount of proteinuria and decreased uric acid excretion.^66^ All these changes may impact drug metabolism in pregnancy, although the extent to which loss of albumin in the urine leads to increased protein bound drug elimination is likely minimal. Clinically, the most relevant change that occurs regarding renal physiology, is an increase in clearance of renally excreted drugs. Renal clearance is broadly the speed with which a substance is removed from plasma and excreted into the urine. Formally, clearance, Cl, is the volume of plasma cleared of a particular drug over unit time, equivalent to (concentration of drug in urine multiplied by the urine flow rate in volume over time), divided by the drug plasma concentration. In general, small uncharged molecules pass easily into the urine and have a high basal renal clearance. In pregnancy, renally excreted drugs will exhibit a higher clearance due to (1) increased renal blood flow, (2) increased GFR, and (3) globally increased activity of renal transporters (including but not limited to organic anion transporters, OAT1, OAT2, and OAT3, as well as organic cation transporter 2, OCT2, and renal P‐glycoprotein, P‐gp), further augmenting clearance of certain small molecules.^67^, ^68^, ^69^

Myriad drugs are affected by pregnancy‐altered renal metabolism and thus an exhaustive list is not practical, however a systematic review of drugs commonly investigated and found to undergo increased renal clearance (and hence at risk of being sub‐therapeutically dosed) include antibiotics, antivirals, antiepileptics, antimalarials, and certain cardiovascular drugs.^70^, ^71^ Notably, both plasma protein bound as well as non‐protein bound drugs are susceptible to hyperfiltration in pregnancy at a given dose. Some “common offenders” of increased renal excretion leading to potential underdosing with significant clinical ramifications include penicillin‐ and cephalosporin‐type antibiotics, SSRIs, the antiepileptic drugs carbamazepine, lamotrigine, levetiracetam, oxcarbazepine, phenytoin, phenobarbital, and topiramate, and the analgesics morphine and ketorolac (although this latter medication, like other nonsteroidal anti‐inflammatory drugs, NSAIDs, is typically avoided in pregnancy).^71^

### Placental Transfer of Medications

Finally, an organ unique to pregnancy and critical in moderating fetal drug passage is the placenta. As with guinea pigs, mice, and non‐human primates, humans have adapted a hemochorial placental strategy, in which maternal blood is separated from fetal blood via a thin, two cell layer membrane comprised of syncytiotrophoblasts and the endothelial cells of fetal capillaries.^72^, ^73^ In general, molecules that are more likely to cross the placental barrier include gases such as CO_2_ and O_2_, glucose, amino acids and fatty acids, water, electrolytes, certain IgG molecules (especially during the second and third trimesters), and certain drugs, particularly those that are of low molecular weight and are uncharged and lipophilic.^74^ Molecules exceeding 500 Da in size rarely pass the placental barrier in the absence of a transporter.^74^ Of note, certain drugs are actively pumped out of fetal circulation via transmembrane transporters such as P‐glycoprotein (MDR1) present in the trophoblast layer.^75^

### Pharmacokinetic Considerations of Therapeutic Peptides and Antibodies

While the discussion thus far has centered around small molecule drugs, consideration must also be given to other classes of pharmaceuticals, including peptides and a special class of proteins—antibodies. Common examples of peptide/protein medications encountered in pregnancy include insulin, oxytocin, and interferons (e.g., interferon‐alpha and interferon‐beta). Physiological adaptations of pregnancy can impact protein medications in meaningful ways. Using insulin as an example, the increased maternal plasma volume and total body water lead to a larger volume of distribution of insulin, which can dilate circulating concentrations and may necessitate higher doses to achieve glycemic targets.^76^ On the other hand, plasma protein–protein interactions are also influenced by pregnancy physiology, with albumin and IgG decreasing in concentration progressively throughout pregnancy in proportion to hemodilution. While interferons are minimally protein bound in plasma, other peptide medications are highly bound to carrier proteins, which can affect their free (unbound) components as pregnancy progresses.^77^ Oxytocin is 60% IgG‐bound, while insulin detemir is 98%–99% albumin bound in plasma; thus their free circulating component will be enriched in the third compared to first and second trimesters as a function of decreased concentrations of IgG and albumin, respectively.^78^, ^79^ A final factor that can influence the pharmacokinetics of protein medications is the lymph flow rate, which is known to increase during pregnancy as an adaptive response to increased blood volume and capillary filtration.^80^ Such increased lymphatic flow can enhance absorption and systemic distribution of peptide medications administered subcutaneously or intramuscularly in pregnancy, leading to higher peak plasma concentrations and faster onset of action.^81^

Regarding antibodies, the IgG subclass is the only subclass of antibodies able to cross the syncytiotrophoblast layer via active transport beginning in the second trimester of pregnancy and increasing thereafter.^82^ Mechanistically, IgG binds FcRn in various tissue types including the placenta, undergoes endocytosis, and enters fetal circulation through the acidified endosome.^82^ Preferential transport occurs for IgG1, followed by IgG4, IgG3, and IgG2, for which the FcRn receptors have the lowest affinity, while certain Fc variants can confer prolonged serum half‐lives of IgG antibodies also due to altered FcRn affinity.^74^, ^82^, ^83^ Regarding their pharmacokinetics, monoclonal antibodies (mAbs) typically have 100% bioavailability, limited tissue distribution due to their molecular weight, are not metabolized by CYP450 enzymes, are eliminated via pinocytosis and lysosomal degradation, and exhibit biphasic elimination with an initial rapid phase followed by a slower phase. A host of therapeutic mAb are used in pregnancy as biologic agents for various autoimmune diseases. Despite their known placental transfer, the anti‐TNF class of mAb have been widely studied and are considered safe in pregnancy.^84^ Other classes of mAb either have insufficient safety evidence due to their recent emergence on the market, or require careful weighing of risks and benefits in pregnancy due to issues such as fetal B cell depletion and lymphopenia, as in the case of rituximab as an example.^85^

The approach we recommend when assessing degree of placental transfer of a given drug is to first refer to any available human data. If human studies are unavailable, the next best resource would be animal model data (preferably those involving animals with a similar placentation strategy to human). If neither of these resources is available, our recommendation is generally to avoid the drug and seek alternatives, unless the benefits to maternal health are anticipated to far exceed theoretical fetal risk (Figure 1). This is especially true during the first trimester of pregnancy during which time organogenesis occurs and at which time consequences of placental transfer of a drug of untested toxicity may be particularly devastating on organ development. Notorious examples of teratogenic drugs previously used in humans in the first trimester include thalidomide, originally marketed in pregnancy for nausea and vomiting but found to cause severe fetal Phocomelia by preventing angiogenic outgrowth of the developing limbs, and valproic acid, known to cause neural tube defects via reactive oxygen species (ROS) production and DNA damage among other mechanisms.^86^, ^87^

**Figure 2:**
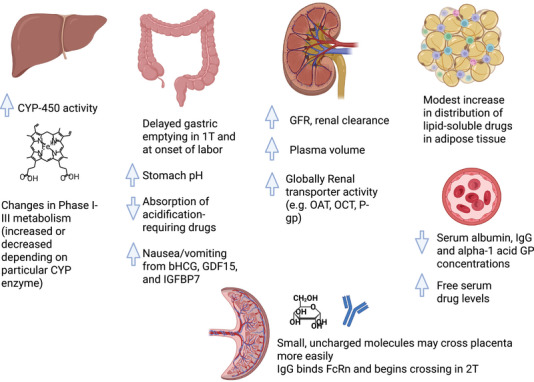
Physiological adaptations of pregnancy that may affect pharmacokinetics. CYP, cytochrome; GFR, glomerular filtration rate; GI, gastrointestinal; GP, glycoprotein.

Figure 2 depicts a summary of major physiological adaptations of pregnancy that may affect pharmacokinetics.

## Conclusions

Here, we have summarized two key aspects of the approach to pharmacotherapy in pregnancy. The first task is to map out the drug's indication, any maternal contraindications or special considerations, then fetal contraindications, using the best available evidence, and then to perform a risk–benefit analysis, weighing maternal and fetal risks and benefits and considering alternatives such as second‐ or third‐line agents as well as the possibility of deferring treatment altogether and opting for close surveillance. This review serves as a call to action to clinicians to utilize the decision tree framework we lay out in Figure 1 to aid in selecting the optimal drug for the maternal or fetal condition. This tool provides a practical, stepwise approach to selecting pharmacotherapy in pregnancy for the busy obstetric provider.

Once a drug is chosen, it is crucial to consider how normal pregnancy physiology may affect pharmacokinetic and pharmacodynamic properties of the drug, as well as to ascertain or estimate transplacental passage, especially for drugs with known or suspected fetal risk. As pregnancy is a dynamic state with fluctuations in weight, volume, and alterations in the functioning of the various organ systems, it is important to consider whether the trimester of pregnancy may be relevant to drug metabolism, as well as to keep trimester in mind when prescribing drugs that may be teratogenic during organogenesis of the first trimester, but may be safer from a fetal standpoint later in pregnancy. Consultation with general or subspecialized (e.g., infectious disease or anticoagulation) pharmacies as well as subspecialist physicians may be warranted to discuss possible dose adjustments, drug monitoring, as well as drug–drug interactions that may impact the mother or fetus. Looking forward, several recently developed classes of medications in the cardiometabolic arena, including the GLP1 receptor agonists and the SGLT2 inhibitors, are becoming more widespread in use among women of reproductive potential. Moreover, emerging therapeutics in the immunomodulatory domain, including chimeric antigen receptor T (CART) cell therapy and other biologic/immunological agents, are entering the pipeline of clinical trials and first‐in‐pregnancy reports. More investigation is needed to understand how these drugs may interact with pregnancy physiology, affect fetal development, and ultimately impact maternal and perinatal outcomes (Supporting Information).

## Funding

This work was supported by National Institutes of Health awards K23HD104517 and DP1HD115433 to ACE and 1U01HD118959‐01 to KW.

## Conflicts of Interest

GWK sits on a Data Safety Monitoring Board for the clinical trial Hyperemesis Gravidarum Risk Reduction with Metformin (NCT07129473). The authors have no other relevant conflicts of interest to report.

## Supporting information



Supporting Information

## Data Availability

This manuscript contains no original data to be shared.
